# Treatment outcomes of pediatric acute myeloid leukemia: a retrospective analysis from 1996 to 2019 in Taiwan

**DOI:** 10.1038/s41598-021-85321-3

**Published:** 2021-03-15

**Authors:** Yung-Li Yang, Tang-Her Jaing, Shih-Hsiang Chen, Hsi-Che Liu, Iou-Jih Hung, Dong-Tsamn Lin, Chao-Ping Yang, Ching-Tien Peng, Kai-Hsin Lin, Chih-Cheng Hsiao, Shiann-Tarng Jou, Jiann-Shiuh Chen, Ming-Tsan Lin, Shih-Chung Wang, Te-Kau Chang, Fang-Liang Huang, Chao-Neng Cheng, Kang-Hsi Wu, Jiunn-Ming Sheen, Shu-Huey Chen, Meng-Yao Lu, Giun-Yi Hung, Hsiu-Ju Yen, Yuh-Lin Hsieh, Jinn-Li Wang, Yu-Hsiang Chang, Hsiu-Hao Chang, Ting-Chi Yeh, Te-Fu Weng, Jen-Yin Hou, Bow-Wen Chen, Rong-Long Chen, Lin-Yen Wang, Wan-Ling Ho, Yu-Chieh Chen, Shin-Nan Cheng, Yu-Hua Chao, Shang-Hsien Yang, Ting-Huan Huang, Shu-Wei Chou, Chien-Yu Lin, Hsuan-Yu Chen, Yu-Mei Y. Chao, Der-Cherng Liang, Tai-Tsung Chang

**Affiliations:** 1 Department of Laboratory Medicine, National Taiwan University Hospital and College of Medicine, National Taiwan University, Taipei, Taiwan; 2 Department of Pediatrics, National Taiwan University Hospital and College of Medicine, National Taiwan University, Taipei, Taiwan; 3 Department of Hematology-Oncology, Chang Gung Children’s Hospital-Linkou and Chang Gung University, Taoyüan, Taiwan; 4 Division of Pediatric Hematology-Oncology, Mackay Memorial Hospital and Mackay Medical College, Taipei, Taiwan; 5 Division of Pediatric Hematology and Oncology, China Medical University Children’s Hospital, Taichung, Taiwan; 6 Department of Biotechnology, Asia University, Taichung, Taiwan; 7 Department of Pediatrics, Chang Gung Memorial Hospital-Kaohsiung Medical Center, Chang Gung University College of Medicine, Kaohsiung, Taiwan; 8 Department of Pediatrics, National Cheng Kung University Hospital, Tainan, Taiwan; 9 Department of Pediatric Hematology & Oncology, Changhua Christian Children’s Hospital, Changhua, Taiwan; 10 Department of Pediatrics, Taichung Veterans General Hospital, Taichung, Taiwan; 11 Department of Pediatrics, Chung Shan Medical University Hospital and School of Medicine, Chung Shan Medical University, Taichung, Taiwan; 12 Department of Pediatrics, Chiayi Chang Gung Memorial Hospital, Chiayi, Taiwan; 13 Department of Pediatrics, Taipei Medical University–Shuang Ho Hospital, Taipei, Taiwan; 14 Department of Pediatrics, Taipei Veterans General Hospital and National Yang-Ming University, Taipei, Taiwan; 15 Taipei Municipal Wan Fang Hospital, Taipei, Taiwan; 16 Department of Pediatrics, Kaohsiung Veterans General Hospital, Kaohsiung, Taiwan; 17 Division of Pediatric Hematology and Oncology, Koo Foundation Sun Yat-Sen Cancer Center, Taipei, Taiwan; 18 Department of Pediatrics, Chi Mei Medical Center, Tainan, Taiwan; 19 School of Medicine, College of Medicine, Fu Jen Catholic University, New Taipei City, Taiwan; 20 Department of Pediatrics, Shin Kong Wu Ho-Su Memorial Hospital, Taipei, Taiwan; 21 Department of Pediatrics, School of Medicine, College of Medicine, Taipei Medical University Hospital, Taipei Medical University, Taipei, Taiwan; 22 Taipei Cancer Center, Taipei Medical University, Taipei, Taiwan; 23 Department of Pediatrics, Tungs’ Taichung MetroHarbor Hospital, Taichung, Taiwan; 24 Department of Pediatrics, Buddhist Tzu Chi General Hospital, Hualien, Taiwan; 25 Institute of Statistical Science Academia Sinica, Taipei, Taiwan; 26 Childhood Cancer Foundation, Taipei, Taiwan; 27 Department of Pediatrics, Kaohsiung Medical University Hospital, Kaohsiung Medical University, Kaohsiung, Taiwan; 28 Department of Pediatrics, Chia-Yi Christian Hospital, No 539, Rhongxiao Road, East Dist., Chia-Yi, 60002 Taiwan

**Keywords:** Medical research, Oncology

## Abstract

Improvement in outcomes of children with acute myeloid leukemia (AML) is attributed to several refinements in clinical management. We evaluated treatment outcomes of Taiwanese pediatric AML patients in the past 20 years. Overall, 860 de novo AML patients aged 0–18 years and registered in the Childhood Cancer Foundation of R.O.C during January 1996–December 2019 were included. Survival analysis was performed to identify factors that improved treatment outcomes. Regardless of treatment modalities used, patients during 2008–2019 had better 5-year event-free survival (EFS) and overall survival (OS) rates than patients during 1996–2007. For patients received the TPOG-AML-97A treatment, only 5-year OS rates were significantly different between patients diagnosed before and after 2008. Patients with *RUNX1*–*RUNX1T1* had similar relapse-free survival rates, but 5-year OS rates were better during 2008–2019. However, the survival of patients who received hematopoietic stem-cell transplantations (HSCT) did not differ significantly before and after 2008. For patients without relapse, the 5-year OS improved during 2008–2019. Non-relapse mortality decreased annually, and cumulative relapse rates were similar. In conclusion, 5-year EFS and OS rates improved during 2008–2019, though intensities of chemotherapy treatments were similar before and after 2008. Non-relapse mortality decreased gradually. Further treatment strategies including more intensive chemotherapy, novel agents’ use, identification of high-risk patients using genotyping and minimal residual disease, early intervention of HSCT, and antibiotic prophylaxis can be considered for future clinical protocol designs in Taiwan.

## Introduction

The survival rate of children with acute myeloid leukemia (AML) has improved significantly over the past 40 years and has reached 70% in recent clinical trials^1–9^. Almost all study groups witnessed improvement in event-free survival (EFS) and overall survival (OS) rates in consecutive trials^1^. Several large recent clinical trials showed that the improvement in outcomes of children with AML is attributed to the refinement of supportive care, the adaptation of therapy according to each patient’s early response to chemotherapy (minimal residual disease [MRD]), disease monitoring techniques, chemotherapy intensification, the introduction of new agents, the selective use of hematopoietic stem-cell transplantation (HCT), and improved salvage therapy^10–12^. Similar to childhood acute lymphoblastic leukemia, pediatric AML now uses risk-directed therapy to improve the clinical outcomes^1,9,13^.

A significant number of patients with childhood AML die of treatment complications, especially infections owing to prolonged and severe neutropenia caused by intensified chemotherapy^10,14–18^. In recent years, most international clinical trials using intensified chemotherapy have attempted to decrease the relapse rate to improve the clinical outcomes. Undoubtedly, the infection rate has increased but the mortality rate has not owing to the improved supportive care, antibiotics prophylaxis, and use of new antibiotic and antifungal agents^10,17,19–24^. Improvement in care provided in the intensive care unit has also improved the final clinical outcomes of pediatric AML patients^25^. In this study, we aimed to assess the differences in clinical outcomes and possible factors contributing to the improved survival of pediatric AML patients in Taiwan.

## Materials and methods

### Patients and protocols

The Childhood Cancer Foundation of R.O.C. (Republic of China) was established in 1982. It provided support, both financial and psychological, to poorly resourced pediatric patients with cancer. While initially it was the first to do so, currently, almost all pediatric hematologists and oncologists (Taiwan Pediatric oncologist group, TPOG) have joined the foundation, and the care of these patients is a cooperative effort. The age at onset for most patients treated by pediatric hematologists and oncologists in Taiwan was less than 18 years. These patients were registered in the Childhood Cancer Foundation of R.O.C. Almost 90% of the AML patients were cared for by doctors who participated in TPOG. In this study, a total of 976 AML patients aged 0–18 years were registered from January 1996 to December 2018.

Patients with secondary AML, myelodysplastic syndrome, acute promyelocytic leukemia, or Down syndrome were excluded from the survival analysis. Patients with AML M3 (Acute promyelocytic leukemia, APL) were treated with TPOG-APL-97 or TPOG-APL-2001. These results will be published separately. A total of 860 newly diagnosed pediatric patients with de novo AML were included in this study. The Institutional Review Board of National Taiwan University Hospital approved the study and all of the participants or their guardians provided informed consent in accordance with the Declaration of Helsinki.

### Diagnosis and classification

The initial diagnosis of AML, including subtyping and immunophenotyping, was performed according to the French–American–British (FAB) classification. The karyotypes were interpreted according to the International System for Human Cytogenetics Nomenclature. Common fusion transcripts such as runt-related transcription factor 1 (*RUNX1–RUNX1T1)* and *CBFB–MYH11* were detected using reverse transcription-polymerase chain reaction (RT-PCR) assays followed by Sanger sequencing in some hospitals. Patients with AML harboring ≥ 3 acquired chromosome aberrations in the absence of prognostically favorable t(8;21)(q22;q22), inv(16)(p13q22)/t(16;16)(p13;q22) and t(15;17)(q22;q21) chromosomal rearrangements were described as having a complex karyotype^26^. Morphological, immunophenotypical, and cytogenetical analyses were carried out at each local hospital. The principal investigator reviewed every case included in this study.

### Treatment

All protocols were approved by the Institutional Review Boards of the participating institutions. Written informed consent was obtained from the patients, parents, or guardians. The TPOG-AML-97A protocol was followed as described previously^27^. We summarize this protocol in Supplementary Figure 1. A further 367 patients received different protocol treatments, i.e., anthracycline, cytarabine, and etoposide in different combinations and doses according to the physicians’ choices.

### Definitions

Complete remission (CR) was defined as trilineage hematopoietic recovery with less than 5% blasts in the marrow. Early death (ED) was defined as death during induction, before CR. Treatment-related mortality (TRM) was defined as any death during the first CR. Refractory disease represented cases that failed to achieve CR after 2 courses of induction therapy, and relapse was defined as disease recurrence after initial CR. Other events included study withdrawal and secondary malignancies. Risk categorization was defined similar to other international trials^1^. Low-risk was defined as patients having t(8;21) *RUNX1-RUNX1T1*, inv(16) *CBFB-MYH11*, or t(9;11) *KMT2A-AF9*. High-risk was defined as patients having one of the following: monosomy 7, t(6;9), M6 or M7 morphology according to FAB, or *FLT3* gene internal tandem duplications. Standard-risk was defined as patents having none of the above.

### Statistical methods

We divided the time range 1996–2019 into two groups to observe the difference in survival. We also divided the entire time period, equally, into four periods to compare survival differences. The primary end points of this study were OS, EFS, and RFS (relapse-free survival). OS was measured from the start of the treatment to death from any cause, and EFS was measured from the start of the treatment to first progression, relapse, or death from any cause. RFS was measured from the date of remission to the that of first relapse only. Patients who did not achieve first remission were assigned an EFS of zero.

Disease-free survival extended from the time of transplantation to the time of its failure. Patients who did not fail were censored at the time of their last follow-up. Survival curves were estimated using the Kaplan–Meier method. The log-rank test was used to assess the survival between different periods. The trend test was used for the analysis of the trends of non-disease mortality. A significant difference was assigned a *p*-value < 0.05. Survival rates are represented as mean percent ± standard deviation probability estimates. All analyses were performed using SAS software, version 9.4 (SAS Institute, Inc., Cary, NC, USA).

## Results

### Patients’ characteristics

The baseline characteristics of the patients in the two periods are stated in Table 1. There were no significant differences in age, sex, white blood cell count, FAB subtypes, or risk categories between the two periods. Characteristics of the patients who received treatment according to the TPOG-AML-97A protocol are described in Table 2.

**Table 1:** Baseline characteristics of patients.

	1996–2007 (n = 505)		2008–2019 (n = 355)		Total (n = 860)		p
	n	% or range	n	%	n	%	
**Gender**							0.487
Male	275	54.46	202	56.9	477	55.47	
Female	230	45.54	153	43.1	383	44.53	
**Age**							0.173
Median, range	8.56	0–18	10.64	0–18	9.48	0–18	
**WBC (k/ml)**							0.016
Median, range	27.36	0.7–726	21.58	0.5–639.4	23.6	0.5–726	
**FAB**							0.008
M0	26	5.15	14	3.94	40	4.65	
M1	65	12.87	43	12.11	108	12.56	
M2	151	29.9	112	31.55	263	30.58	
M4	80	15.84	62	17.46	142	16.51	
M5	62	12.28	53	14.93	115	13.37	
M6	18	3.56	8	2.25	26	3.02	
M7	54	10.69	39	10.99	93	10.81	
M8	0	0	1	0.28	1	0.12	
M9	20	3.96	21	5.92	41	4.77	
Unknown	29	5.74	2	0.56	31	3.6	
**Chromosome**							< 0.0001
-5/del(5q)/-7/del(7q)	11	2.18	8	2.25	19	2.21	
11q23	14	2.77	12	3.38	26	3.02	
Complex	34	6.73	32	9.01	66	7.67	
N/A	127	25.15	26	7.32	153	17.79	
Normal	130	25.74	91	25.63	221	25.7	
Other	83	16.44	76	21.41	159	18.49	
inv(16)	17	3.37	20	5.63	37	4.3	
t(6;9)	4	0.79	0	0	4	0.47	
t(8;21)	74	14.65	69	19.44	143	16.63	
t(9;11)	8	1.58	20	5.63	28	3.26	
t(9;22)	3	0.59	1	0.28	4	0.47	

FAB: French–American–British, WBC: white blood cell.

**Table 2:** Baseline characteristics of patients who received the TPOG-97A treatment.

	1996–2007 (n = 231)		2008–2019 (n = 266)		total (n = 497)		p
	n	%	n	%	n	%	
**Gender**							0.240
Male	122	52.81	155	58.27	277	55.73	
Female	109	47.19	111	41.73	220	44.27	
**Age**							< 0.0001
Median, range	6.94	0.01–17.86	11.05	0–18	9.03	0–18	
**WBC**							0.147
Median, range	27.18	1.07-549.30	21.74	0.5-635	23.40	0.5-635	
**FAB**							0.082
M0	15	6.49	11	4.14	26	5.23	
M1	31	13.42	37	13.91	68	13.68	
M2	82	35.5	88	33.08	170	34.21	
M4	40	17.32	52	19.55	92	18.51	
M5	30	12.99	46	17.29	76	15.29	
M6	11	4.76	4	1.5	15	3.02	
M7	21	9.09	20	7.52	41	8.25	
M9	1	0.43	8	3.01	9	1.81	
**Chromosome**							0.016
-5/del(5q)/-7/del(7q)	6	2.6	4	1.5	10	2.01	
11q23	13	5.63	11	4.14	24	4.83	
Complex	18	7.79	21	7.89	39	7.85	
N/A	29	12.55	10	3.76	39	7.85	
Normal	62	26.84	77	28.95	139	27.97	
Other	44	19.05	55	20.68	99	19.92	
inv(16)	10	4.33	15	5.64	25	5.03	
t(6;9)	2	0.87	0	0	2	0.4	
t(8;21)	38	16.45	58	21.8	96	19.32	
t(9;11)	6	2.6	14	5.26	20	4.02	
t(9;22)	3	1.3	1	0.38	4	0.8	

FAB: French–American–British, WBC: white blood cell.

### Five-year EFS and OS rates according to the FAB classification and cytogenetical analysis

**Figure 1:**
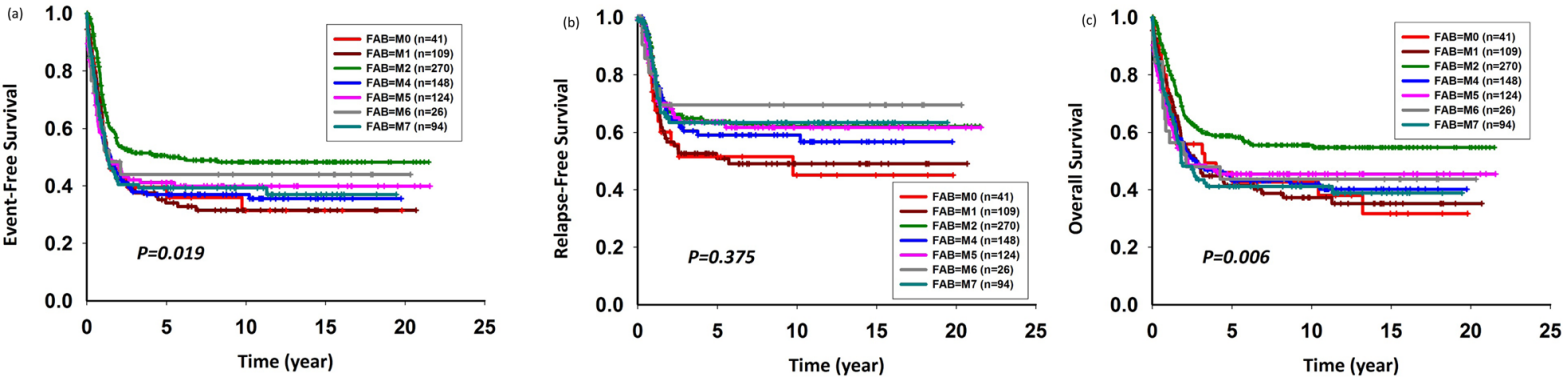
Five-year (**a**) event-free survival, (**b**) relapse-free survival, and (**c**) overall survival rates according to the French–American–British classification.

**Figure 2:**
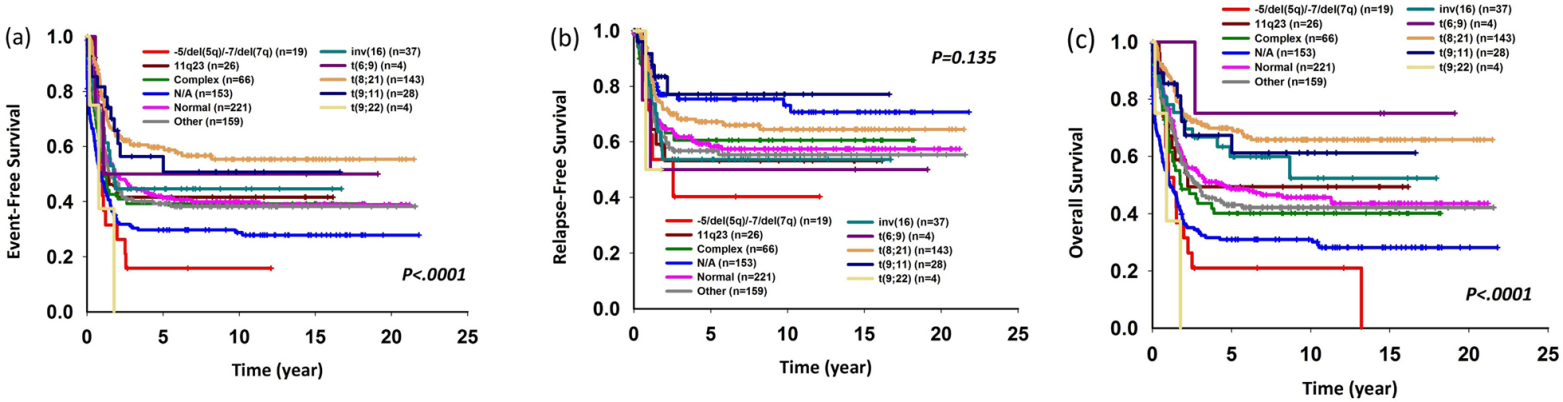
Five-year (**a**) event-free survival, (**b**) relapse-free survival, and (**c**) overall survival rates according to major cytogenetic alterations.

The 5-year EFS and OS per the FAB classification and major cytogenetic alterations demonstrated by the entire cohort are described in Figs. 1 and 2. Patients with FAB subtypes M2 and M4 had better outcomes than patients with FAB subtypes M7 and M6. Patients with t(8;21) and inv (16) cytogenic changes had better outcomes. Patients with *BCR-ABL1* or complex cytogenetic changes had poor outcomes.

### Five-year EFS, OS, cumulative incidence of relapse rates and non-relapse OS for the time periods 1996–2007 and 2008–2019

**Figure 3:**
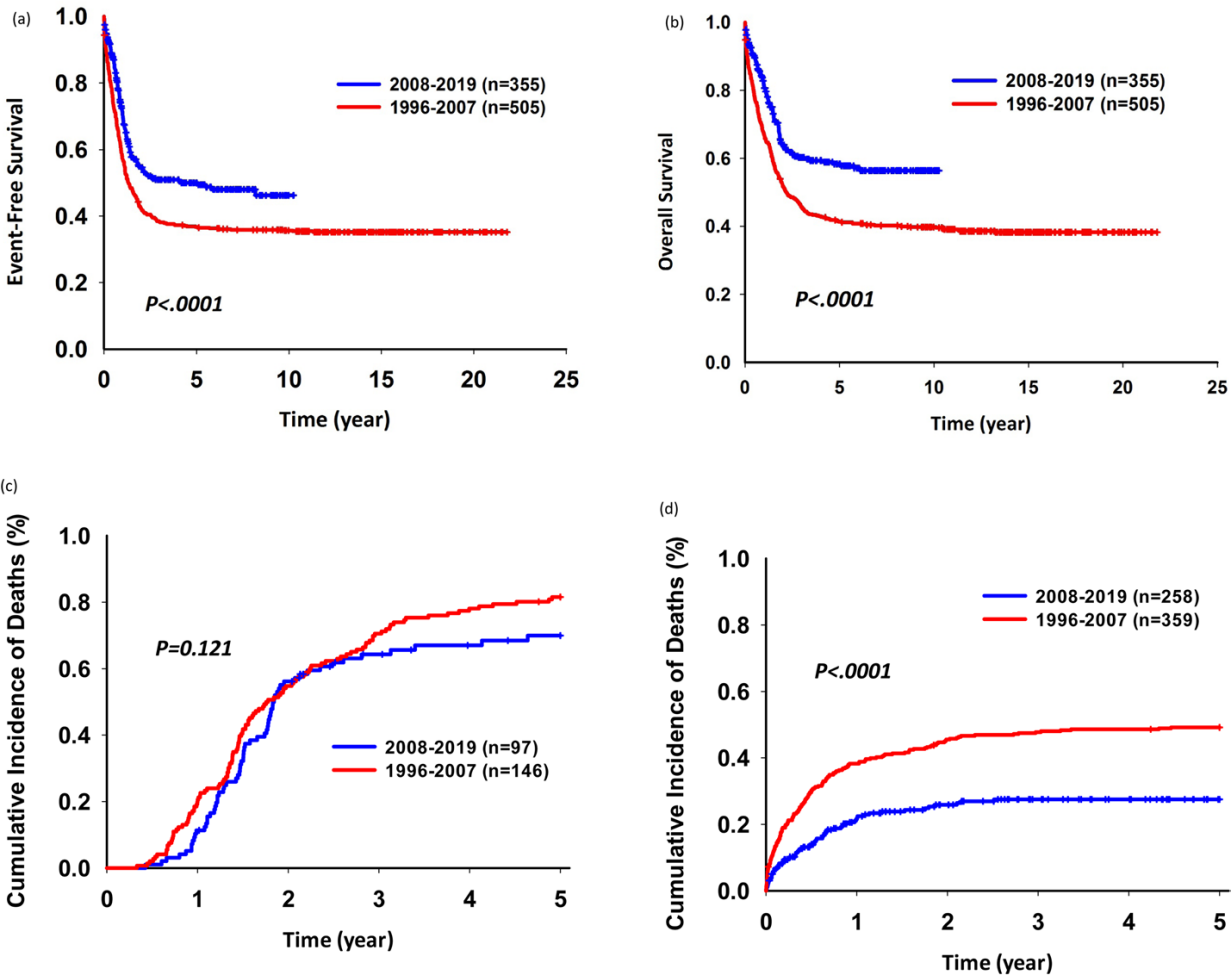
Five-year (**a**) event-free survival, (**b**) overall survival, and (**c**) cumulative incidence of relapse rates (**d**) non-relapse overall survival of patients of the entire cohort during 1996–2007 and 2008–2019.

We analyzed the 5-year EFS and OS rates before and after 2008 to assess if these outcomes improved over time. The 5-year EFS rate was better in patients diagnosed after 2008 than before 2008 (49.99%; 95% confidence interval [CI], 44.23–55.48 vs. 36.67%; 95% CI 32.47–40.88; *p* < 0.001) (Fig. 3). The 5-year OS rate was better in patients diagnosed after 2008 than before 2008 (58.39%; 95% CI 52.56–63.77 vs. 41.42; 95% CI 37.09–45.68; *p* < 0.001). In addition, the cumulative incidence of the relapse rate was similar between these two periods. However, the non-relapse death rate improved for patients during 2008–2019 (Fig. 3).

### Five-year EFS and OS rates of patients treated according to the TPOG-AML-97A protocol

The 5-year EFS rate was better in patients diagnosed after 2008 than before 2008, but the difference was insignificant (52.71%; 95% CI 45.94–59.02 vs. 46.69%; 95% CI 40.14–52.97; *p* = 0.144) (Supplementary Figure 2). The 5-year OS rate was better in patients diagnosed after 2008 than before 2008 (61.63; 95% CI 54.75–67.78 vs. 51.86; 95% CI 45.22–58.08; *p* = 0.016). In addition, the cumulative incidence of relapse rate was similar between these two periods. However, the non-relapse death rate improved for patients during 2008–2019 (Supplementary Figure 2).

### Five-year EFS and OS rates of low-risk patients with *RUNX1-RUNX1T1*

Assessments of improvements in patients with *RUNX1-RUNX1T1* showed that they had similar RFS rates in both the periods (72.62; 95% CI 58.67–83.87 vs. 73.49; 95% CI 53.90–85.77; *p* = 0.582). However, the 5-year EFS (57.89; 95% CI 40.75–71.69 vs 71.01; 95% CI 56.32–81.54; *p* = 0.183) and OS (63.16; 95% CI 45.86–76.27 vs. 83.13; 95% CI 70.05–90.85; p = 0.019) rates were better for patients diagnosed after 2008 than before 2008, but the difference was not significant for the 5-year EFS rate (Supplementary Figure 3).

### Outcomes of patients without relapse

**Figure 4:**
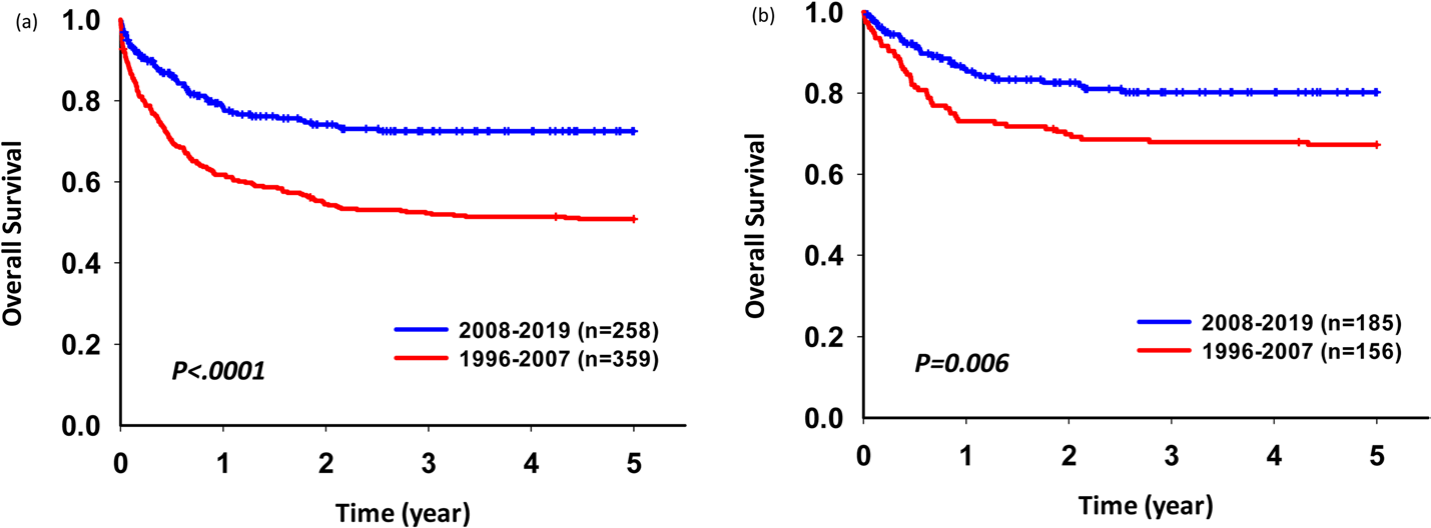
Five-year overall survival rates for non-relapse patients. (**a**) entire cohort (**b**) patients receiving the TPOG-AML-97A treatment protocol.

**Figure 5:**
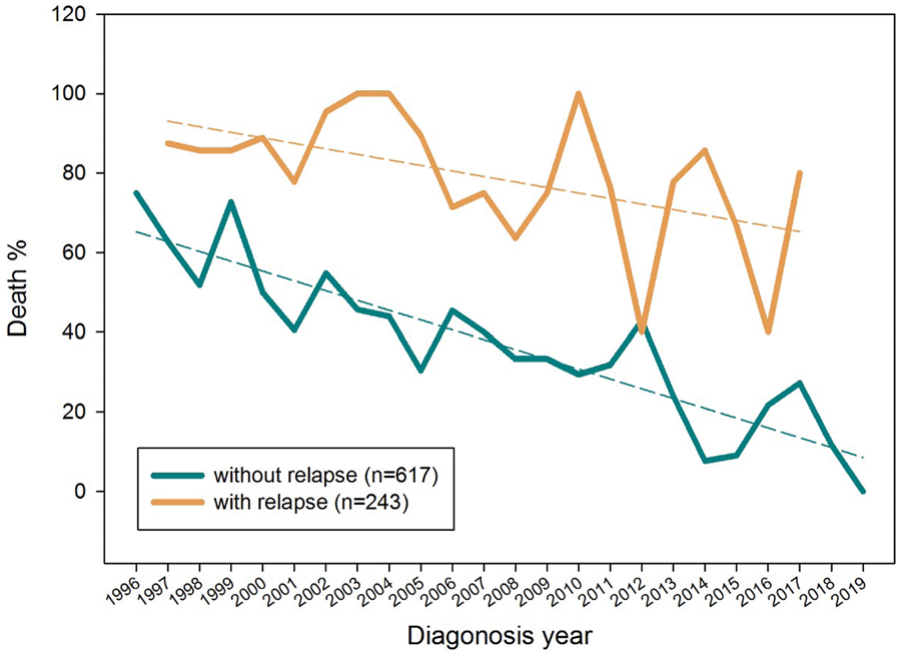
A gradual decrease in the percentage of non-disease mortality over time.

In 617 patients without relapse, the 5-year OS rate was poorer during 1996–2007 than during 2008–2019 (50.81% vs. 72.51%, p < 0.001). For patients who received treatment according to the TPOG-97A protocol, the trend of the 5-year OS rate was the same (Fig. 4). With time, mortality owing to non-relapse, decreased gradually (Fig. 5).

### Outcomes after hematopoietic stem-cell transplantation (HSCT)

The 5-year EFS and OS rates after HSCT between these two periods did not differ significantly (Supplementary Figure 4). The donor types of HSCT is listed in Table 3. Autologous HSCTs were seldom performed after 2008. More patients, including those with CR1 and a relapsed disease status, received HSCTs after 2008 (Table 3). The results of our analysis of the clinical characteristics of CR1 patients in these two periods are shown in Supplementary Table 1. For patients with *RUNX1-RUNX1T1* or *CBFB-MYH11*, the results of HSCTs were better than for patients with other AML subtypes, though only the 5-year OS rate had significance (p = 0.038). The relapsed patients’ findings also showed a similar trend (Supplementary Figure 5).

**Table 3:** Time point of HSCT between two periods.

	1996–2007 (n = 505)		2008–2019 (n = 355)		Total (n = 860)		p value
	N	%	N	%			
**HSCT timing**							0.026
After relapse	35	6.93	40	11.27	75	8.72	
CR1	90	17.82	75	21.13	165	19.19	
No	380	75.25	240	67.61	620	72.09	
**Donor type (n = 240)**							
Matched Sibling	40	32.00	36	31.31	76	31.67	< 0.001
Unrelated	22	17.60	61	53.04	83	34.58	
Autologous	17	13.60	1	0.87	18	7.50	
Cord blood	6	4.80	3	2.61	9	3.75	
Haplo-identical	4	3.20	12	10.43	16	6.67	
Unknown	36	28.80	2	1.74	38	15.83	

HSCT: Hematopoietic stem-cell transplantation; CR: complete remission.

### Five-year EFS and OS rates of patients during 2013–2019 were better than those of other patients in other periods

**Figure 6:**
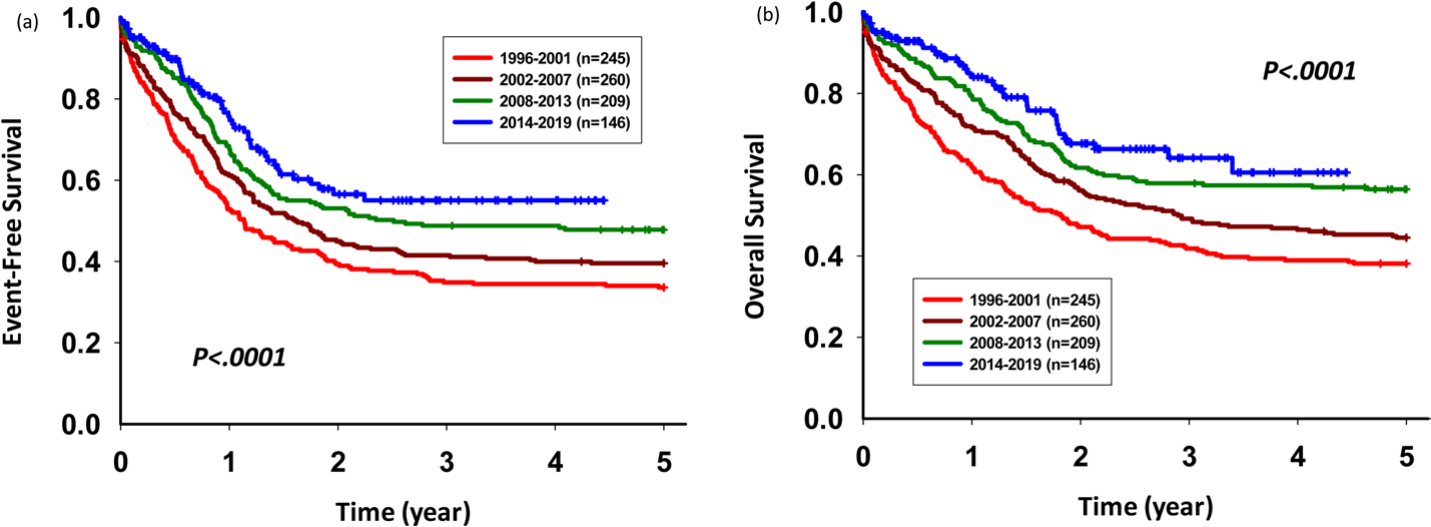
Five-year (**a**) event-free survival (EFS) and (**b**) overall survival (OS) rates based on the four periods. The 5-year EFS and OS increased over time.

We further divided the 1996–2019 time range for the entire cohort into four periods, 1996–2001, 2002–2007, 2008–2012, and 2013–2019. The 5-year EFS rate for each period was as follows: 33.61, 95% CI 27.76–39.59; 39.55, 95% CI 33.59–45.45; 47.83, 95% CI 40.91–54.41; 55.05, 95% CI 44.71–64.23, *p* < 0.001. The 5-year- OS was rate was follows: 38.12, 95% CI 32.04–44.17; 44.51, 95% CI 38.39–50.44; 56.42, 95% CI 49.42–62.83; 60.57, 95% CI 47.97–71.02, *p* < 0.001. The 5-year EFS and OS rates of patients during 2013–2019 was better than those of other patients in other periods (Fig. 6).

## Discussion

Although chemotherapy regimens for childhood AML have not changed significantly over the past 20 years, our study found that the 5-year EFS and OS rates improved after 2008 in Taiwan. The improved treatment outcomes may be attributed to the improved survival of non-relapsed patients. The survival after HSCTs and relapse rate did not differ significantly between the two periods. Supportive care, including antifungal and antibiotic prophylaxis, has improved the outcomes in some hospitals^19^. For patients without relapse, the cause of death owing to infection or other causes decreased significantly, leading to an increase in the 5-year EFS and OS after 2008. For patients with relapse, HSCTs improved the survival of low-risk patients, such as those with *RUNX1-RUNX1T1*, but not the outcomes for other relapsed patients. Non-disease mortality has decreased gradually over the past 20 years, suggesting an improvement in pediatric oncological supportive care.

Over the past 20 to 30 years, the trend of improved pediatric AML treatment outcomes in major international clinical trials was significant^1,10–12^. Patients with *RUNX1-RUNX1T1* benefited from being participants in several consecutive clinical trials, which consisted of intensive therapies based on either higher dose anthracycline or high-dose cytarabine^10,28–30^. Thomas et al. showed that patients with inv(16) also demonstrated the same trend in St. Jude trials, though the significance was not prominent owing to a small sample size^10^. This improvement reflects differences in the treatment intensity between the protocols in the different periods. However, unlike in these trials, chemotherapy regimens did not change significantly in Taiwan.

Considering patients with *RUNX1-RUNX1T1*, the 5-year RFS was almost the same but the 5-year OS improved significantly after 2008, probably owing to the use of salvage treatment such as HSCT after disease relapse. Hu et al*.* also showed that HSCT can improve the prognosis of high-risk pediatric t(8;21) AML based on MRD-guided treatment^31^. The 5-year EFS rate of patients with *RUNX1-RUNX1T1* was 10–20% lower in this cohort than in other recent clinical trials^2,32–34^. Increasing the dose of chemotherapy might improve clinical outcomes of low-risk patients, such as those with *RUNX1-RUNX1T1*, thus preventing them from receiving HSCTs during disease relapse.

Several large clinical trials have shown that TRM and ED decreased over time, but chemotherapy intensity has increased. Bochennek et al. compared two AML-BFM protocols. Infection-related morbidity was slightly higher when the AML-BFM 2004 protocol was used than when the AML-BFM 93 protocol was used (3.3 vs. 2.8 infections per patient, respectively), whereas infection-related mortality decreased significantly when the former protocol was used (1.5% vs. 5.4%, respectively; *p* = 0.003)^17^. Specific anti-infective recommendations were included in the treatment protocol; regular training courses for pediatric hematologists and increased experience in patient care may also be reasons for the reduced infection-related mortality of children with AML in AML-BMF studies^21,35^. Inaba et al*.* used antibiotic prophylaxis to reduce sepsis in pediatric AML patients^36^. Yeh et al*.* used a similar protocol in a Taiwanese medical center and reported a reduced infection-related mortality^19^. Antibiotic prophylaxis for all AML patients may improve outcomes by decreasing infection-associated mortality in Taiwan^19,21,35^.

The findings of several international clinical trials can provide guidelines for future protocol design. The investigators from St Jude Children’s Hospital used MRD risk-directed therapy in AML02. Gemtuzumab-ozogamicin (GO) was used to treat patients with high MRD. The 3-year EFS and OS rates were 63.0% ± 4.1% and 71.1% ± 3.8%, respectively^2^. To decrease the possibility of cardiac toxicity and secondary leukemia, the results of trials on AML 08 indicated the possibility of clofarabine replacing anthracycline and etoposide^3^. GO was added to the standard chemotherapy in Children’s Oncology Group trial AAML0531. GO can improve the EFS rate due to reducing the relapse rate^8^. There were several strategies from which we could learn. One was using MRD to identify patients who may not respond to chemotherapy well and who may need intensification with other agents. Another was to use second-line chemotherapy such as clofarbine instead of the first-line chemotherapy. However, these patients could not opt for novel therapies available for the treatment of pediatric AML owing to financial constraints^2,37,38^. While the National Insurance Company has covered the expenses of pediatric cancer treatment since 1995, thereby reducing the treatment abandonment rates of pediatric cancer patients in Taiwan, its disadvantage lie in the lack of cover for novel therapies. Therefore, the inclusion of novel treatment agents in the cover provided by the National Insurance Company should be considered in future Taiwanese pediatric AML clinical trials.

Another hurdle in the improvement of survival of pediatric AML is the high-risk patients. A significant difference in the overall cumulative incidence of relapsed disease was not observed between the two periods in this study. This was expected as the intensity of chemotherapy did not change significantly over the past 20 years in Taiwan. Even if the intensity of chemotherapy increased, the relapse or refractory rate of high-risk patients did not differ significantly in several clinical pediatric AML trials. MRD is another way to identify high-risk patients and is helpful to improve clinical outcomes^2,4,34^. Rasche et al. analyzed the AML-BFM trials from 1987 to 2012 and concluded that further intensification of anthracycline-based and cytarabine-based chemotherapy will most likely not cause an eminent reduction in the number of relapsing or refractory patients^11^. High-risk patients still had dismal outcomes despite receiving HSCTs^11,39^. Novel treatment or immunotherapy might be indicated instead^8,37,38,40–46^.

Our study had several limitations. Since a retrospective study design was used, causality between the intervention and outcomes could not be determined. Our analysis of ED and TRM was confounded by supportive-care measures that evolved over time, and the principles of these measures may have varied in different hospitals. There was no standard salvage protocol for relapsed patients, and this may have affected the remission status of patients before HSCTs and, subsequently, would have impacted the final results. This issue may be resolved in future prospective study designs through the incorporation of supportive-care elements, including antibiotic and antifungal prophylaxis.

In conclusion, although chemotherapy dose intensities did not increase significantly, the 5-year EFS and OS rates improved after 2008. The main cause of treatment failure remained disease relapse. Designing a more intensive chemotherapy regimen should be considered in Taiwan for low-risk AML patients to decrease their relapse rates. However, to identify high-risk patients, sequencing efforts and determining MRD levels after induction should be considered, which in turn would help in the timely referral of these patients for early HSCTs or other novel therapies^2,3,37–39,47–49^. In addition, antibiotic prophylaxis for this subset of patients should be considered by the National Insurance Company to decrease mortality or morbidity caused by intensified chemotherapy.

## Supplementary Information

Supplementary Information.
