# Supplementary material for: Treatment outcomes of pediatric acute myeloid leukemia: a retrospective analysis from 1996 to 2019 in Taiwan
Source: Sci Rep. 2021 Mar 15;11:5893. doi: 10.1038/s41598-021-85321-3 (PMC7960737; doi:10.1038/s41598-021-85321-3)
Supplement: Supplementary file 1 — Supplementary Information. [file 41598_2021_85321_MOESM1_ESM.pdf]

Treatment outcomes of pediatric acute myeloid leukemia: A retrospective analysis from 1996 to 2019 in Taiwan

Yung-Li Yang[1,2], Tang-Her Jaing[3], Shih-Hsiang Chen[3], Hsi-Che Liu[4], Iou-Jih Hung[3], Dong-Tsamn Lin[1,2], Chao-Ping Yang[3], Ching-Tien Peng[5,6], Kai-Hsin Lin[2], Chih-Cheng Hsiao[7], Shiann-Tarng Jou[2], Jiann-Shiuh Chen[8], Ming-Tsan Lin[9], Shih-Chung Wang[9], Te-Kau Chang[5], Fang-Liang Huang[10], Chao-Neng Cheng[8], Kang-Hsi Wu[11], Jiunn-Ming Sheen[7,12], Shu-Huey Chen[13], Meng-Yao Lu[2], Giun-Yi Hung[14], Hsiu-Ju Yen[14], Yuh-Lin Hsieh[14], Jinn-Li Wang[15], Yu-Hsiang Chang[16], Hsiu-Hao Chang[2], Ting-Chi Yeh[4], Te-Fu Weng[11], Jen-Yin Hou[4], Bow-Wen Chen[17], Rong-Long Chen[17], Lin-Yen Wang[18], Wan-Ling Ho[19,20,21,], Yu-Chieh Chen[7], Shin-Nan Cheng[22], Yu-Hua Chao[11], Shang-Hsien Yang[23], Ting-Huan Huang[4], Shu-Wei Chou[2], Chien-Yu Lin[24], Hsuan-Yu Chen[24], Yu-Mei Y. Chao[25], Der-Cherng Liang[4#], Tai-Tsung Chang[26,27*]

[1]Department of Laboratory Medicine, National Taiwan University Hospital and College of Medicine, National Taiwan University, Taipei, Taiwan

[2]Department of Pediatrics, National Taiwan University Hospital and College of Medicine, National Taiwan University, Taipei, Taiwan

[3]Department of Hematology-Oncology, Chang Gung Children's Hospital-Linkou and Chang Gung University, Taoyuan, Taiwan

[4]Division of Pediatric Hematology-Oncology, Mackay Memorial Hospital and Mackay Medical College, Taipei, Taiwan

[5]Division of Pediatric Hematology and Oncology, China Medical University Children's Hospital, Taichung, Taiwan

[6]Department of Biotechnology, Asia University, Taichung, Taiwan

[7]Department of Pediatrics, Chang Gung Memorial Hospital-Kaohsiung Medical Center, Chang Gung University College of Medicine, Kaohsiung, Taiwan

[8]Department of Pediatrics, National Cheng Kung University Hospital, Tainan, Taiwan

[9]Department of Pediatric Hematology & Oncology, Changhua Christian Children’s Hospital

[10]Department of Pediatrics, Taichung Veterans General Hospital, Taichung, Taiwan

[11]Department of Pediatrics, Chung Shan Medical University Hospital and School of Medicine, Chung Shan Medical University, Taichung, Taiwan

[12]Department of Pediatrics, Chiayi Chang Gung Memorial Hospital, Chiayi, Taiwan

[13]Department of Pediatrics, Taipei Medical University–Shuang Ho Hospital, Taipei, Taiwan

[14]Department of Pediatrics, Taipei Veterans General Hospital and National Yang-Ming University, Taipei, Taiwan

[15]Taipei Municipal Wan Fang Hospital, Taipei, Taiwan

[16]Department of Pediatrics, Kaohsiung Veterans General Hospital, Kaohsiung, Taiwan

[17]Division of Pediatric Hematology and Oncology, Koo Foundation Sun Yat-Sen Cancer Center, Taipei, Taiwan

[18]Department of Pediatrics, Chi Mei Medical Center, Tainan, Taiwan

[19]School of Medicine, College of Medicine, Fu Jen Catholic University, New Taipei City, Taiwan

[20]Department of Pediatrics, Shin Kong Wu Ho-Su Memorial Hospital, Taipei, Taiwan

[21]Department of Pediatrics, School of Medicine, College of Medicine, Taipei Medical University, Taipei, Taiwan; Taipei Cancer Center, Taipei Medical University, Taipei, Taiwan; Department of Pediatrics, Taipei Medical University Hospital, Taipei, Taiwan

[22]Department of Pediatrics, Tungs' Taichung MetroHarbor Hospital, Taichung, Taiwan

[23]Department of Pediatrics, Buddhist Tzu Chi General Hospital, Hualien, Taiwan

[24]Institute of Statistical Science Academia Sinica, Taipei, Taiwan

[25]Childhood Cancer Foundation, Taipei, Taiwan

[26]Department of Pediatrics, Kaohsiung Medical University Hospital, Kaohsiung Medical University, Kaohsiung, Taiwan

[27]Department of Pediatrics, Chia-Yi Christian Hospital, Chia-Yi 60002, Taiwan

[#]Deceased, Professor Der-Cherng Liang. Dr Der-Cherng Liang was listed as co-corresponding author with Dr Tai-Tsung Chang

Corresponding author: *Tai-Tsung Chang

Department of Pediatrics, Chia-Yi Christian Hospital, No 539, Rhongxiao Road, East Dist., Chia-Yi 60002 Taiwan; Email: taits.chang@gmail.com; Telephone: +886-5-2765041; Fax: +886-5-2788421

Induction

Idarubicin (9 mg/m^2/day × 3) and Cytarabibe (100 mg/m^2/day × 7)

Idarubicin (9 mg/m^2/day × 3) and Cytarabibe (100 mg/m^2/day × 7)

or

Mitoxantrone (8 mg/m^2/day × 5) and Etoposide (100 mg/m2/day × 5) every 4 weeks if remission is not achieved with the above chemotherapy regimen

Consolidation

Cytarabibe (1 g/m^2/12 h on days 1–4) and Etoposide (100 mg/m^2/day × 5)

Cytarabibe (1 g/m^2/12 h on days 1–4) and Mitoxantrone (10 mg/m^2/day on days 2–5)

Cytarabibe (1 g/m^2/12 h on days 1–4) and Etoposide (100 mg/m^2/day × 5)

Cytarabibe (1 g/m^2/12 h on days 1–4) and Mitoxantrone (10 mg/m^2/day on days 2–5)

Post-remission 4 cycles

Idarubicin (9 mg/m^2/day × 1) and Cytarabibe (200 mg/m^2/day × 5) followed

Supplementary Figure 1. Summary of TPOG-AML-97A chemotherapy

(a)

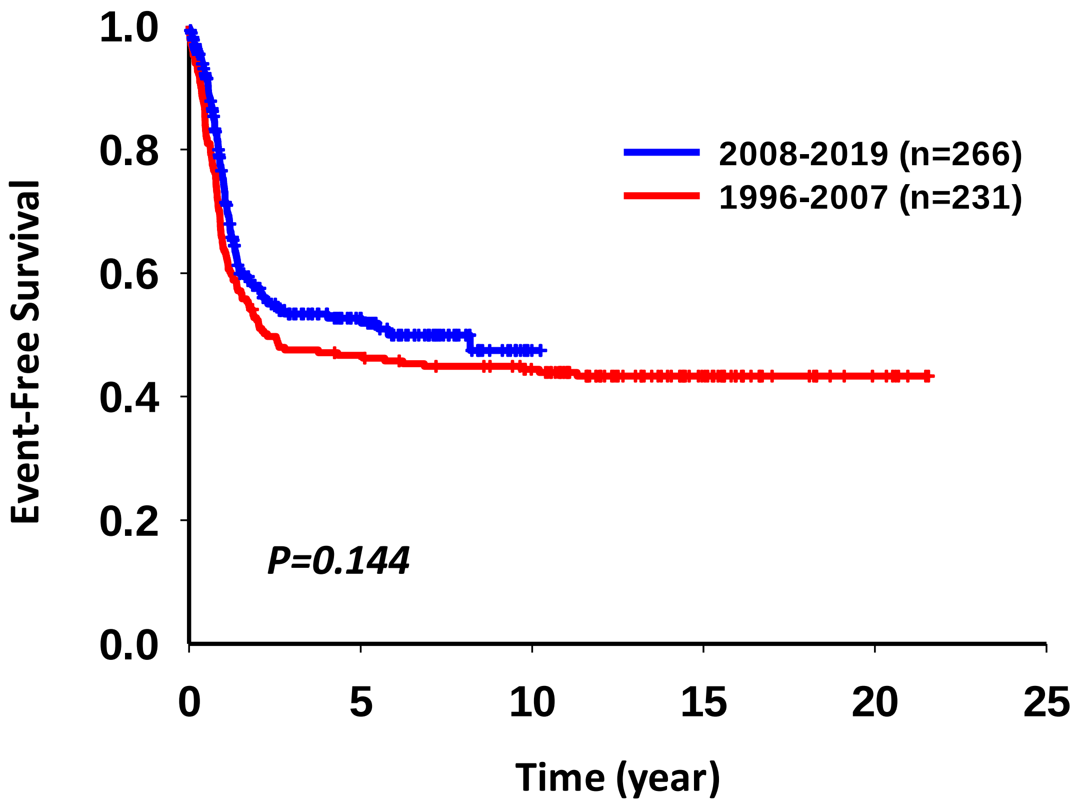

Event-Free Survival
1.0
0.8
0.6
0.4
0.2
0.0
2008-2019 (n=266)
1996-2007 (n=231)
P=0.144
0
5
10
15
20
25
Time (year)

(b)

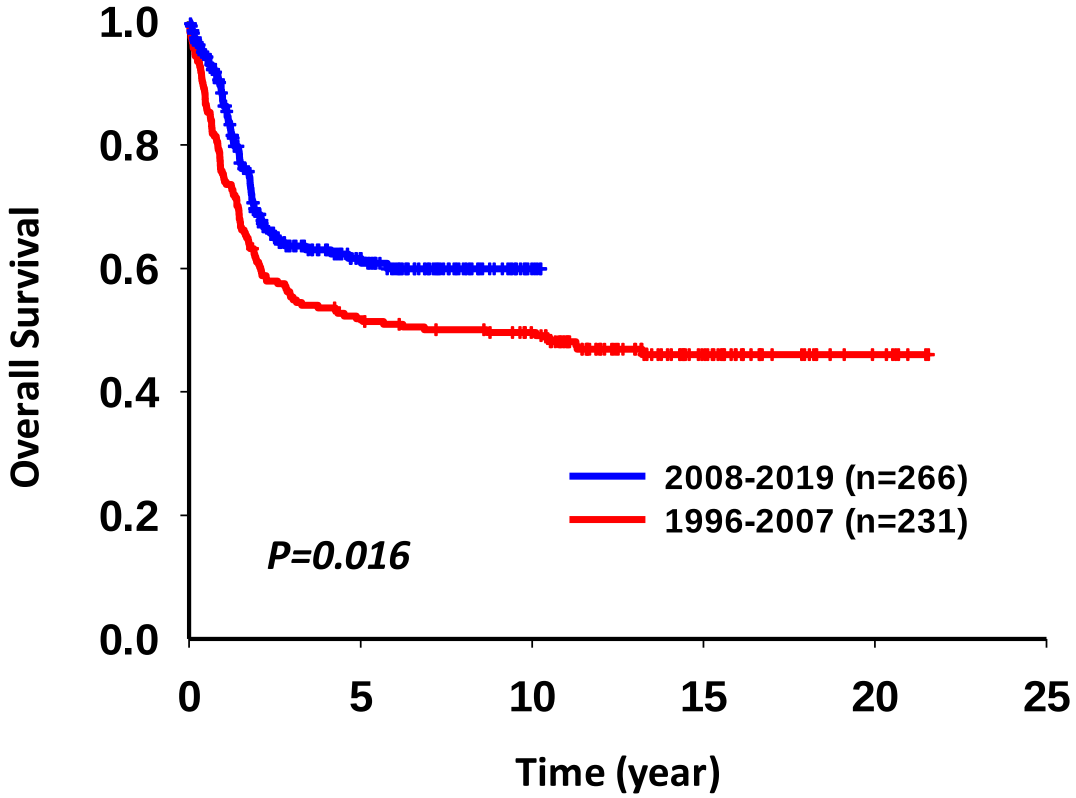

1.0
0.8
0.6
0.4
0.2
0.0
Overall Survival
2008-2019 (n=266)
1996-2007 (n=231)
P=0.016
0
5
10
15
20
25
Time (year)

(c)

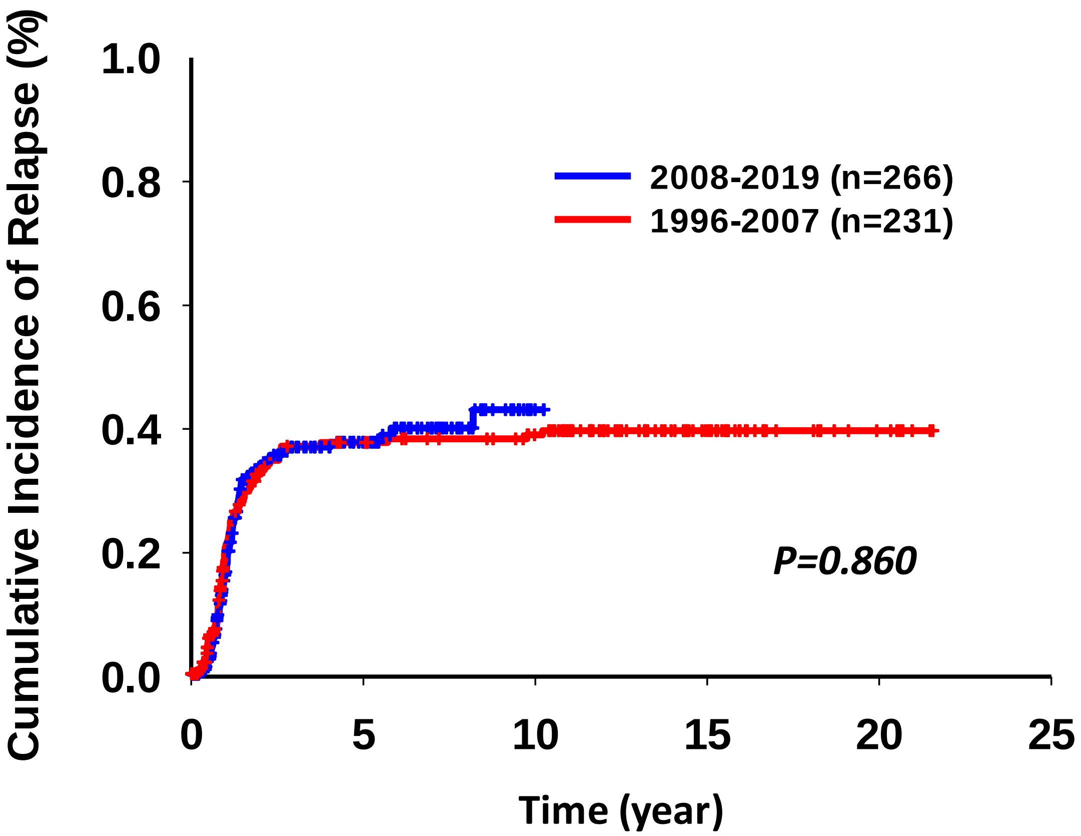

Cumulative Incidence of Relapse (%)
1.0
0.8
0.6
0.4
0.2
0.0
2008-2019 (n=266)
1996-2007 (n=231)
P=0.860
0
5
10
15
20
25
Time (year)

(d)

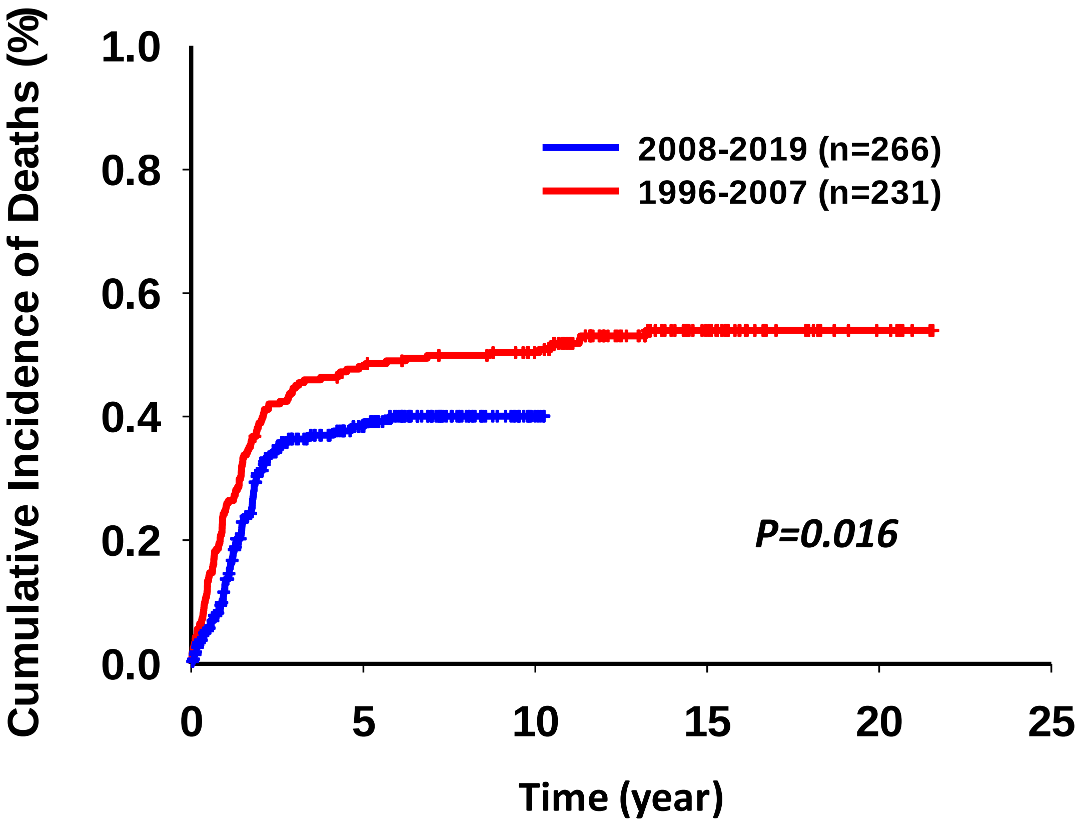

Supplementary Figure 2. Five-year (a) event-free survival, (b) overall survival, (c) cumulative relapsed rates, and (d) non-relapse overall survival of patients who received the TPOG-AML-97A treatment protocol during 1996–2007 and 2008–2019.

(a)

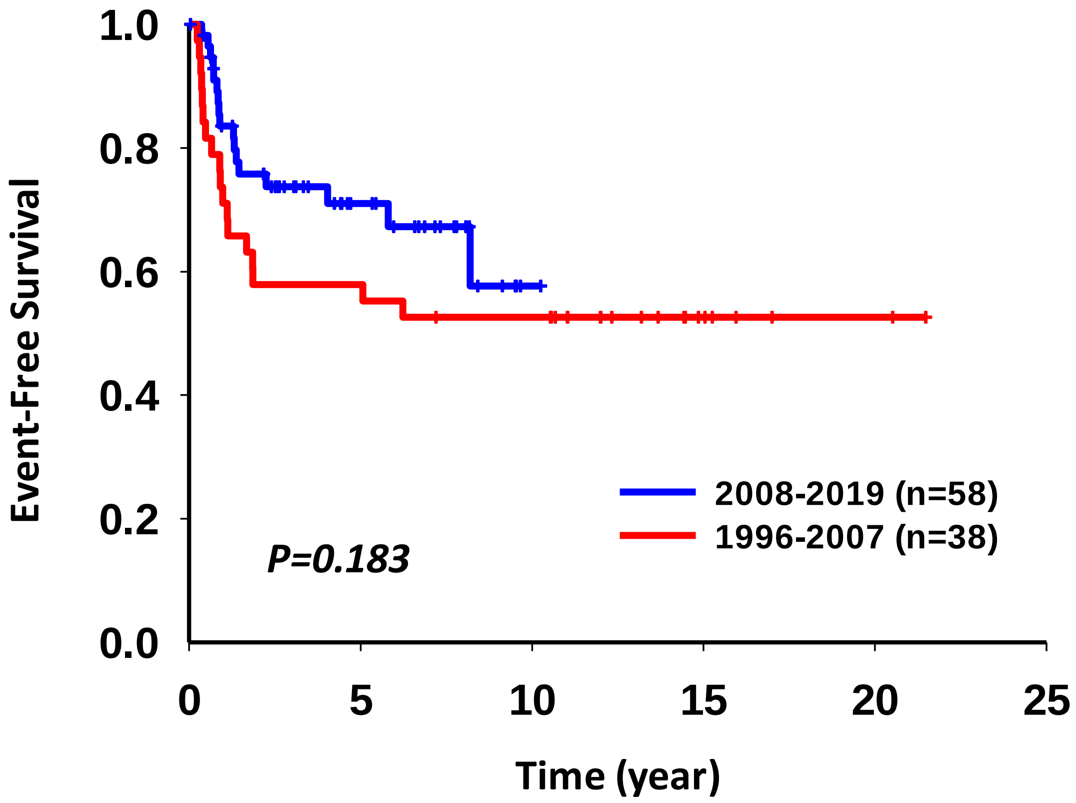

Event-Free Survival
1.0
0.8
0.6
0.4
0.2
0.0
0
5
10
15
20
25
Time (year)
2008-2019 (n=58)
1996-2007 (n=38)
P=0.183

(b)

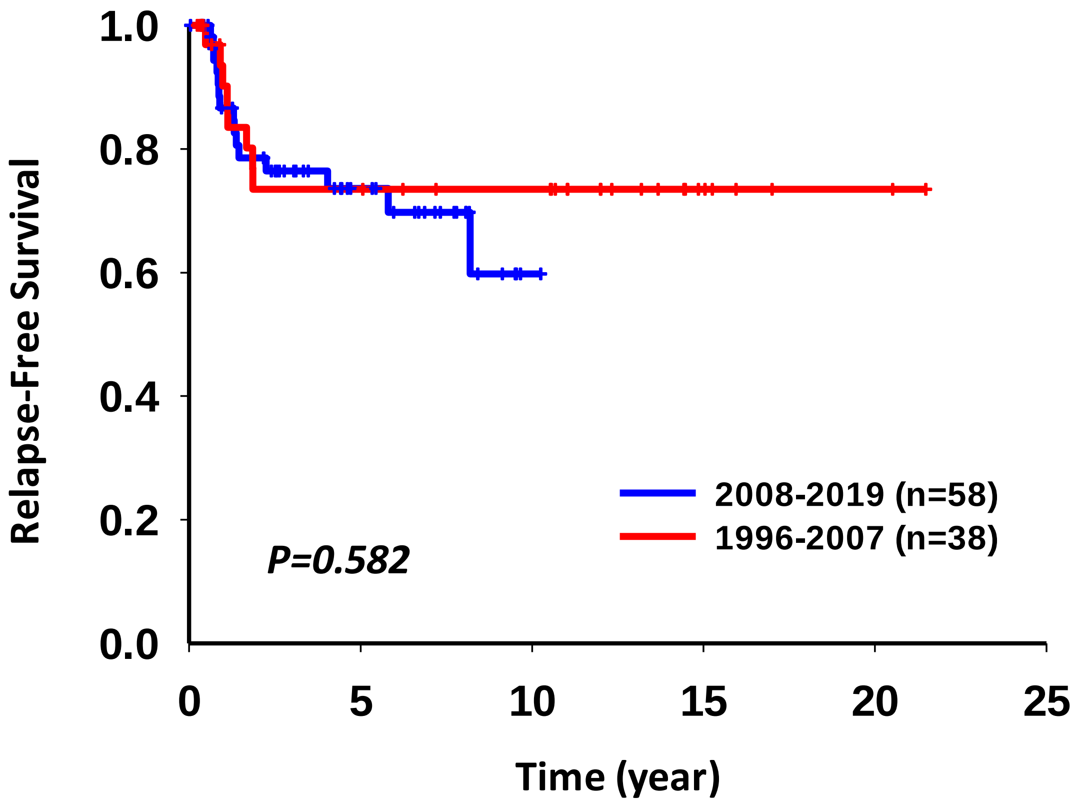
1.0
0.8
0.6
0.4
0.2
0.0
Relapse-Free Survival
P=0.582
2008-2019 (n=58)
1996-2007 (n=38)
0
5
10
15
20
25
Time (year)

(c)

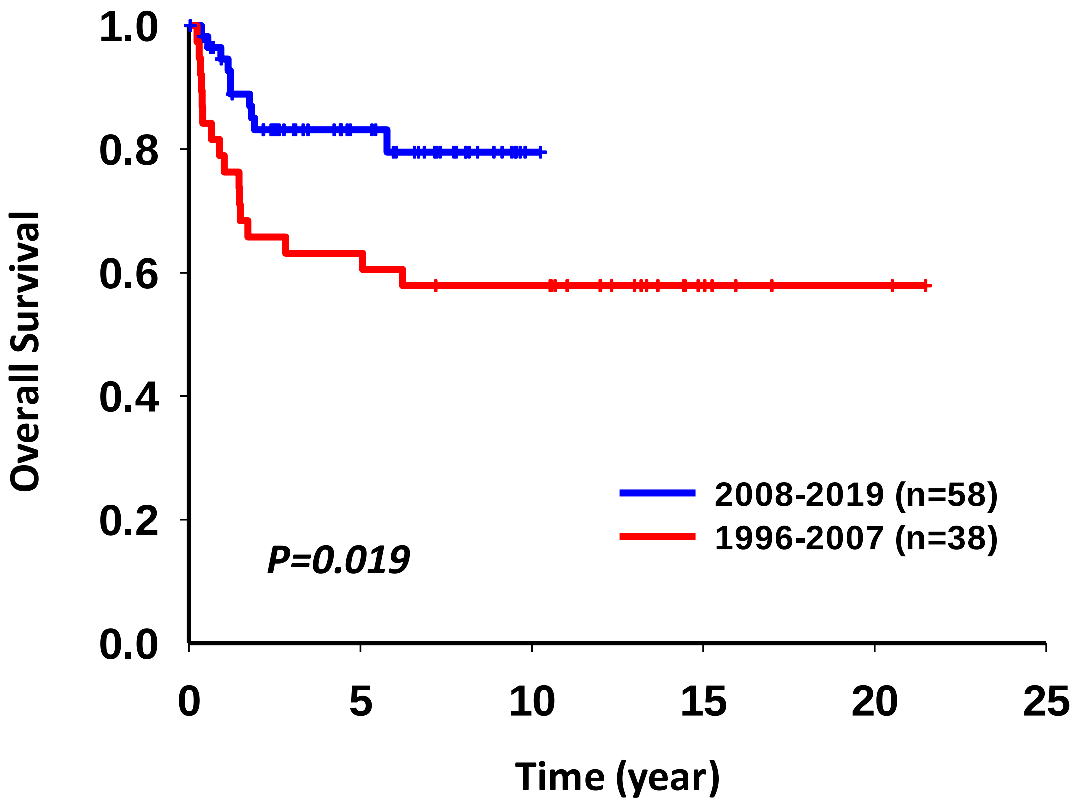

Supplementary Figure 3. 5-year (a) event-free survival, (b) relapse-free survival, and (c) overall survival rates of patients with *RUNX1–RUNX1T1*.

(a)

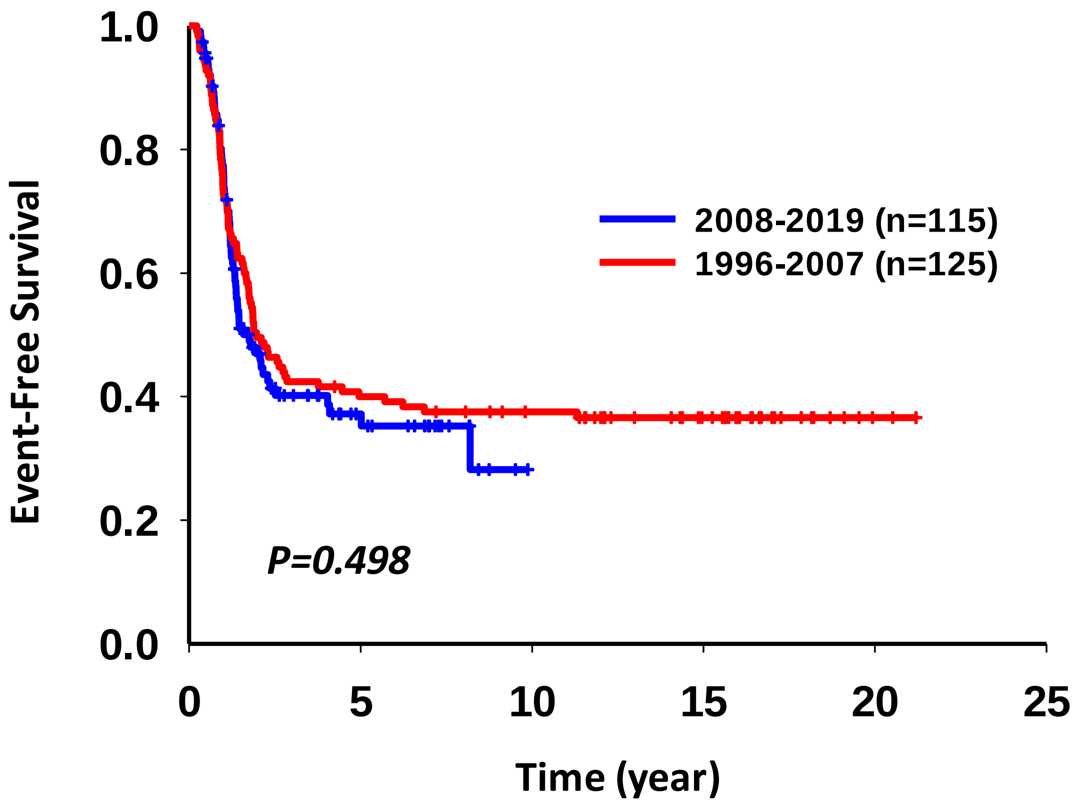

1.0
0.8
0.6
0.4
0.2
0.0
Event-Free Survival
2008-2019 (n=115)
1996-2007 (n=125)
P=0.498
0
5
10
15
20
25
Time (year)

(b)

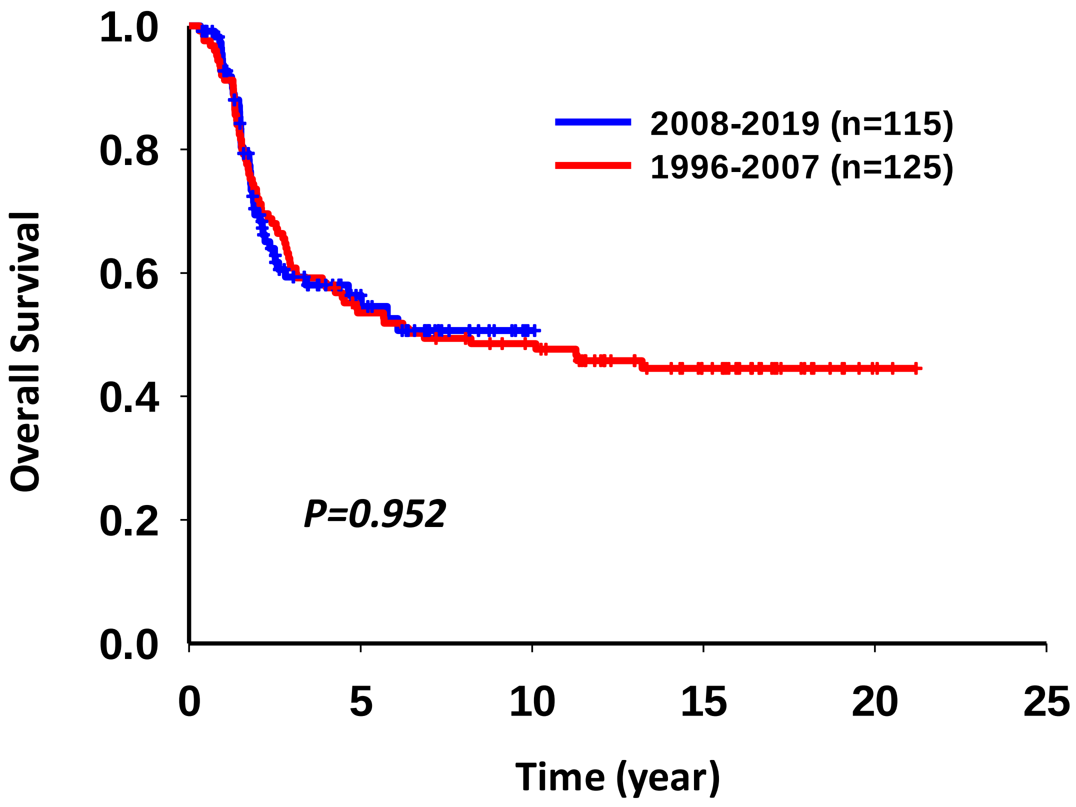

Supplementary Figure 4. Non-significant differences between the 5-year (a) event-free survival and (b) overall survival rates between the two periods

(a)

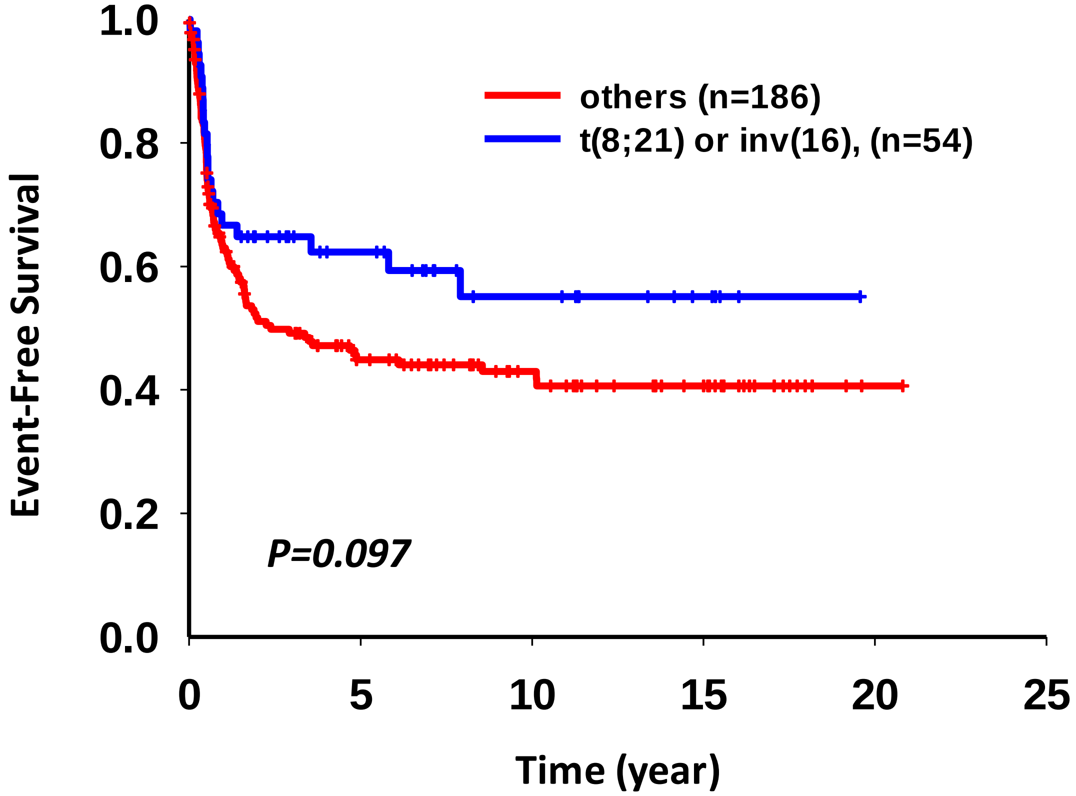

1.0
0.8
0.6
0.4
0.2
0.0
Event-Free Survival
others (n=186)
t(8;21) or inv(16), (n=54)
P=0.097
0
5
10
15
20
25
Time (year)

(b)

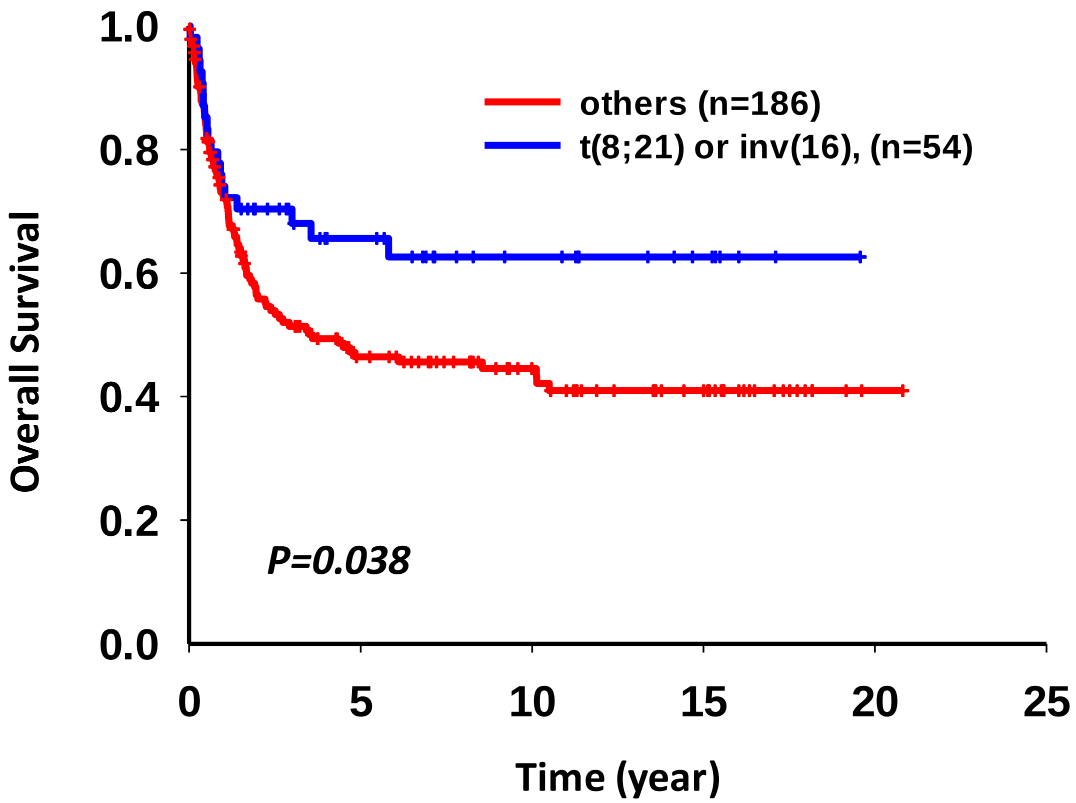

1.0
0.8
0.6
0.4
0.2
0.0
Overall Survival
others (n=186)
t(8;21) or inv(16), (n=54)
P=0.038
0
5
10
15
20
25
Time (year)

(c)

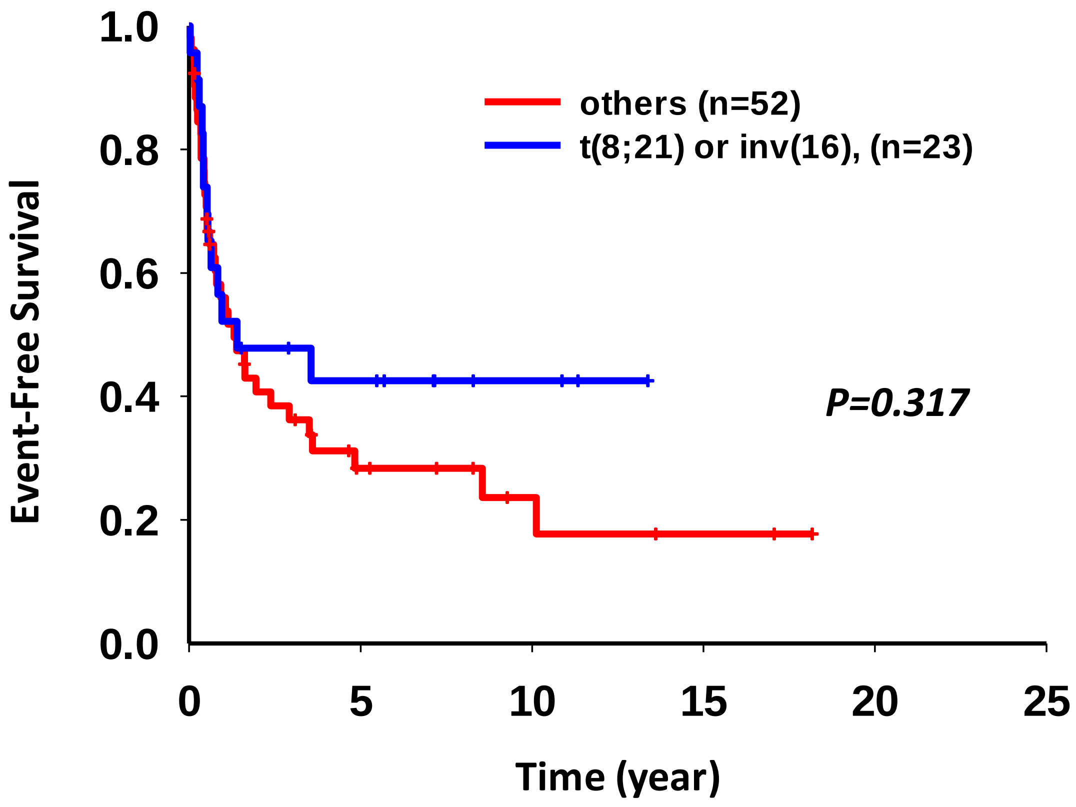
1.0
0.8
0.6
0.4
0.2
0.0
Event-Free Survival
others (n=52)
t(8;21) or inv(16), (n=23)
P=0.317
0
5
10
15
20
25
Time (year)

(d)

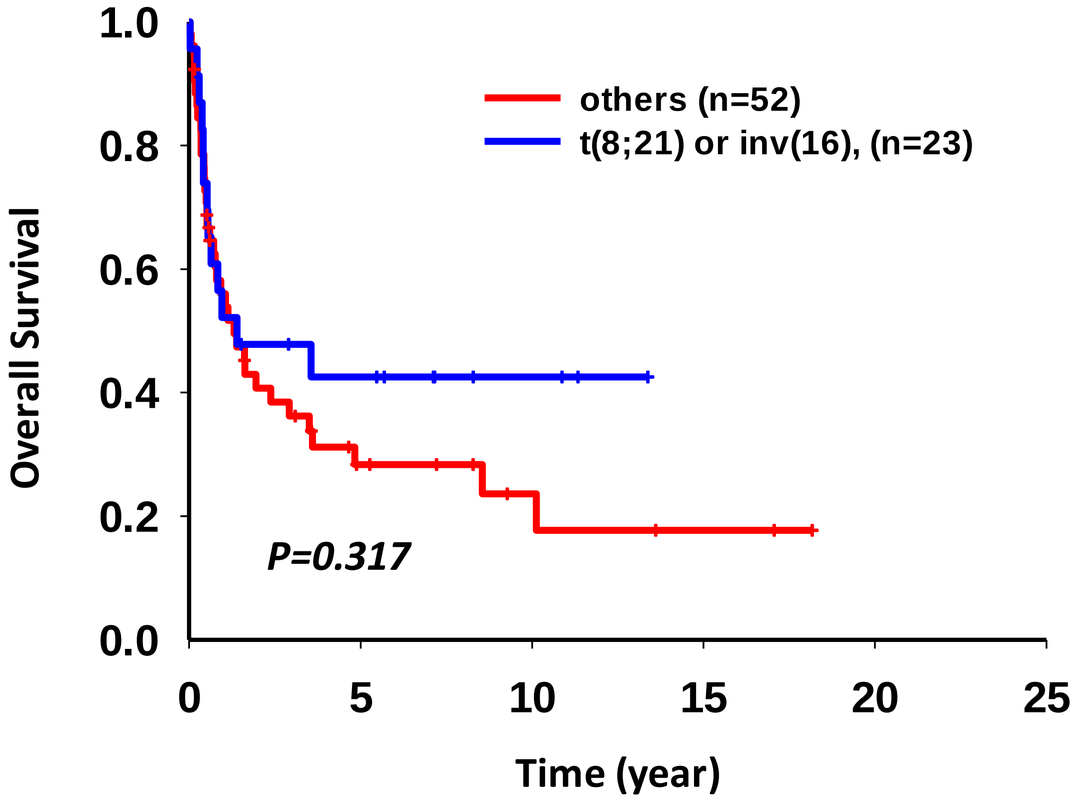

Supplementary Figure 5. (a)(b) Patients with *RUNX1–RUNX1T1* or *CBFB–MYH11* have better outcomes after bone marrow transplantions. (c)(d) For patients with relapse followed by bone marrow transplantions, the trend is the same.

Supplementary Table 1. Baseline characteristics of patients in CR1 who received HSCTs

	1996–2007 (n=90)		2008–2019 (n=75)		Total (n=165)		
	n	%	n	%	n	%	p
Gender							0.057
Male	46	51.11	50	66.67	96	58.18	
Female	44	48.89	25	33.33	69	41.82	
Age							0.991
Median, range	11.59	0.01-17.99	12.10	0.09-17.75	11.88	0.01-17.99	
WBC							0.988
Median, range	24.55	0.7-549.30	23.18	0.80-435.60	23.60	0.7-549.30	
FAB							0.553
M0	5	5.56	6	8	11	6.67	
M1	13	14.44	11	14.67	24	14.55	
M2	35	38.89	25	33.33	60	36.36	
M4	12	13.33	10	13.33	22	13.33	
M5	8	8.89	11	14.67	19	11.52	
M6	3	3.33	1	1.33	4	2.42	
M7	7	7.78	6	8	13	7.88	
M9	3	3.33	5	6.67	8	4.85	
Unknown	4	4.44	0	0	4	2.42	
Chromosome							0.025
-5/del(5q)/-7/ del(7q)	2	2.22	2	2.67	4	2.42	
11q23	1	1.11	4	5.33	5	3.03	
Complex	6	6.67	7	9.33	13	7.88	
N/A	15	16.67	2	2.67	17	10.3	
Normal	34	37.78	20	26.67	54	32.73	
Other	11	12.22	18	24	29	17.58	
inv(16)	1	1.11	0	0	1	0.61	
t(6;9)	1	1.11	0	0	1	0.61	
t(8;21)	15	16.67	15	20	30	18.18	
t(9;11)	3	3.33	7	9.33	10	6.06	
t(9;22)	1	1.11	0	0	1	0.61	

Abbreviations: FAB: French–American–British, WBC: white blood cell, CR: complete remission
